# Extensive Base of Skull Osteomyelitis: Why Does It Still Occur?

**DOI:** 10.7759/cureus.51560

**Published:** 2024-01-03

**Authors:** Siti Hajar Noordiana, Emilia Rosniza Mohammed Rusli, Zara Nasseri, Asma Abdullah

**Affiliations:** 1 Otorhinolaryngology, Universiti Kebangsaan Malaysia Medical Centre, Cheras, MYS; 2 Otorhinolaryngology, Hospital Sungai Buloh, Selangor, MYS; 3 Radiology, Universiti Kebangsaan Malaysia Medical Centre, Cheras, MYS; 4 Otolaryngology, Universiti Kebangsaan Malaysia Medical Centre, Cheras, MYS

**Keywords:** malignant otitis externa, intracranial infection, otorrhea, skull-base osteomyelitis, base of the skull

## Abstract

Skull base osteomyelitis (SBO) is a rare yet serious intratemporal infection that often masquerades as a skull base malignancy. It is most common in diabetic and immunocompromised patients. We present a case of an elderly diabetic patient with end-stage renal disease with progressive malignant otitis externa. The disease progressed to involve the base of the skull, causing multiple cranial neuropathies. Early initiation of intravenous (IV) antibiotics, along with supportive treatment, may improve the long-term prognosis of the disease. This case highlights the importance of keeping a high index of diagnostic suspicion for SBO in patients with risk factors. Early diagnosis and prompt treatment can drastically decrease morbidity and mortality due to SBO.

## Introduction

Skull base osteomyelitis (SBO) is a severe and often fatal infection of the bone and surrounding tissues at the base of the skull. It may be caused by bacterial, fungal, or other infectious agents and usually involves the temporal, sphenoid, or occipital bones [1]. Meltzer and Keleman first coined the term ‘skull base osteomyelitis’ in 1959 to describe patients with pyocyaneus chondritis and osteomyelitis of the external auditory canal [2]. It is a rare clinical entity whose incidence is difficult to determine due to its variable and non-specific clinical presentation. However, its incidence has increased over the past few years, presumably due to more advanced diagnostics tools and an increased number of susceptible individuals [3].

SBO usually is an unfortunate complication of an improperly treated middle ear, nasal, or dental infection [2,4]. The diagnosis and treatment of SBO are particularly challenging for physicians. Its non-specific symptoms, protracted clinical course, and appearance of an infiltrative lesion on imaging may cause it to be mistaken for malignancies [1,2]. The key to diagnosing SBO is keeping a high index of clinical suspicion, especially in diabetic patients or immunocompromised patients presenting with recurrent malignant otitis externa [1]. The common presenting symptoms include a deep, throbbing, and persistent temporal headache accompanied by deep-seated otalgia with different presentations of otorrhea (mucopurulent, scanty, foul smelling, etc.), facial palsy, fever, difficulty swallowing, and other neurological manifestations [4].

Imaging modalities, such as computed tomography (CT) scans and magnetic resonance imaging (MRI), are first-line investigations. However, it is often difficult to distinguish an SBO from its differentials, for example, nasopharyngeal carcinoma, skull base metastasis, clival chondroma, or pituitary macroadenoma based on imaging [1,5]. Hence the investigation of choice is a histopathological examination of the biopsied specimen followed by its culture and sensitivity to identify the causative microorganisms [1]. Pseudomonas aeruginosa and methicillin-resistant Staphylococcus aureus (MRSA) are the most common causative agents. It is rarely caused by fungi such as Aspergillus or Mucor [1,4].

The treatment regimen is based on administering IV broad-spectrum antibiotics and surgical debridement in specific cases. Surgical debridement is necessary if there is abscess formation, extensive soft tissue infiltration, or in refractory cases [4,5]. New clinical guidelines stress the importance of combining hyperbaric oxygen therapy with the conventional treatment regimen [6,7]. The overall survival rates for SBO are approximately 90% and 57% for 18 months and 3 years, respectively [4-6]. However, in the diabetic population, mortality rates increase by 21% to 70% [6]. Hence, adequate control of risk factors, i.e., blood sugar levels in this case, is crucial to improving prognosis [8].

## Case presentation

A 65-year-old male, who was diabetic and hypertensive with end-stage renal disease, presented with excruciating left-sided otalgia and otorrhea for one month. His diabetes was uncontrolled, and he was on regular hemodialysis. Clinically, there was no facial nerve weakness. The left ear extended to the mandibular ramus, the mastoid tip was tender, and the ear canal was filled with necrotic granulation tissue. He was initially diagnosed with left necrotizing otitis externa (NOE). He was prescribed IV ceftazidime 2 grams every 8 hours for 12 days. This was subsequently changed to oral ciprofloxacin 750 mg BD for one month.

**Figure 1:**
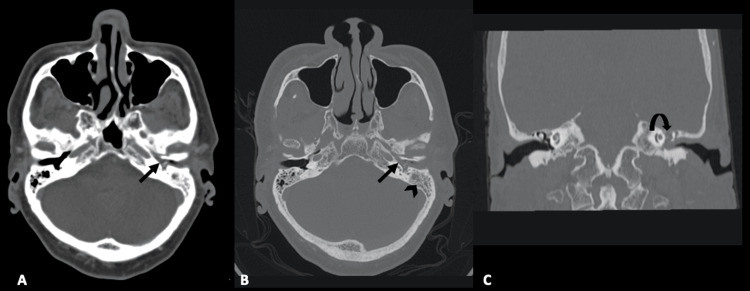
HRCT temporal in (A) axial soft tissue window, (B) axial bone window, and (C) coronal bone window Presence of fluid density within the external and middle ear cavities (long arrow) as well as mastoid air cells (arrowhead) indicating left otitis externa, left otitis media, and left mastoiditis. The normal left tympanic membrane is not visualized. There is a dehiscence of the left tegmen tympani (curved arrow) seen in image (C), suggestive of bony erosion. HRCT: high-resolution computed tomography

There were no signs of abscess collection in the external auditory canal, middle ear, inner ear, or mastoid region. A provisional diagnosis of left chronic otitis media (COM) with intratemporal complications, i.e., facial nerve palsy, was considered, and cortical mastoidectomy was planned. The intraoperative microscopic findings showed a posterior wall sagging with an edematous external auditory canal obscuring the tympanic membrane. The middle ear was filled with granulation tissue, dehiscence at the tegmen mastoidi with granulation overlying granulation tissue. No pus or granulation tissue was seen in the mastoid air cells, and the mastoid bone had a non-sclerotic glue-like appearance. No signs of erosion were seen at the incus and malleolus. The facial nerve was non-responsive to stimulation; it was tested until 3 milliAmperes. Figure 1 shows the initial high-resolution computed tomography (HRCT).

**Figure 2:**
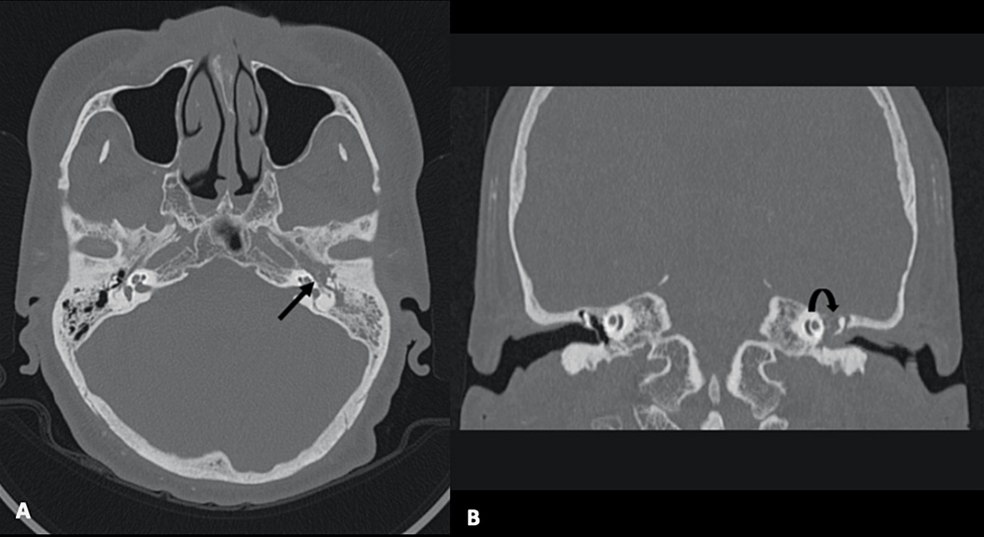
HRCT temporal in (A) axial and (B) coronal views taken about one month apart, demonstrating erosion of the middle ear cavity (long arrow) and worsening dehiscence of the left tegmen tympani HRCT: high-resolution computed tomography

**Figure 3:**
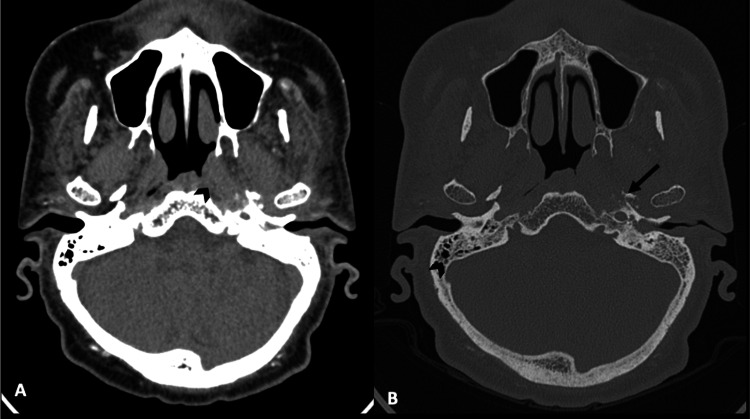
HRCT temporal (A) in soft tissue and (B) in bone windows shows a fullness of the left fossa of Rosenmuller (arrowhead) with associated erosion of the left base of the skull HRCT: high-resolution computed tomography

The patient was nursed postoperatively in the ward and later discharged home. He presented again three months later with recurrent left otalgia and otorrhea. It was associated with headaches and poor oral intake leading to dehydration. HRCT temporal bone was repeated (Figure 2 and Figure 3), which revealed a constellation of CT features that were suggestive of base of the skull osteomyelitis with post-surgical changes of the left mastoid bone. Preoperatively, he was also given a differential diagnosis of nasopharyngeal carcinoma (NPC).

He underwent mastoid exploration, and oto-endoscopic intraoperative findings revealed a generalized edematous external auditory canal with the posterosuperior wall sagging and intact but a pulsatile tympanic membrane. There was no pus or discharge. Mastoid exploration revealed that the middle ear was full of granulation tissue extending to the eustachian tube. The mastoid segment and tympanic segment of the facial nerve were identified and preserved physiologically on nerve stimulation. There was no facial canal dehiscence.

A biopsy of the left nasopharyngeal mass was taken for histopathological examination (HPE). HPE was reported as granulation tissue, with no evidence of malignancy. Postoperatively, the patient was well. He completed a course of IV ceftriaxone 1 gram for 21 days and was discharged home with oral ciprofloxacin 750 mg for one month.

The patient presented yet again three months later with similar complaints of left otalgia and otorrhea. His complaints were the same as previously. It was affecting his sleep and oral intake. The left ear discharge was minimal, yellowish, without foul smell or blood. There was no history of retro-orbital pain, vertigo, tinnitus, vomiting, or seizures. He also complained of loss of appetite and weight loss, about 10 kg within the past few months. He had an aspiration of liquids. On examination, he had a lethargic and toxic look with left facial nerve palsy, House Brackmann grade 2, trismus, absent gag reflex, and sensory loss of the left half of the face. Nystagmus was absent. The left ear examination showed a well-healed postauricular scar and no swelling or tenderness over the mastoid area. The tympanic membrane showed a small central perforation over the posteroinferior quadrant, with no attic retraction, keratin debris, or granulation tissue seen. There were no signs of posterior wall sagging or pus discharge. The right ear was normal. Blood investigations showed high white blood cells (12x10^9/L), and C-reactive protein was raised (14 mg/dL). The erythrocyte sedimentation rate test was high (40 mm/hr), and random blood sugar was 14 mmol/L.

**Figure 4:**
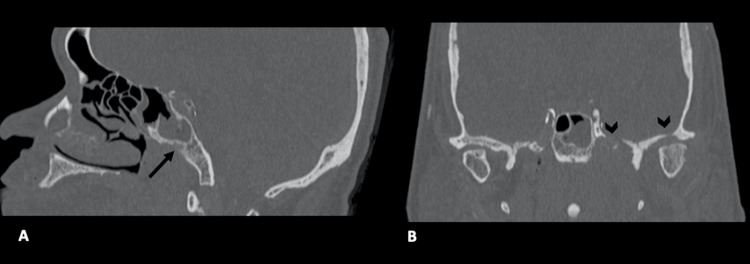
HRCT of temporal (A) in sagittal and (B) coronal views three months later show persistent fullness in the posterior nasopharynx with clival erosion (long arrow). There are also worsening bony erosions at the left base of the skull (arrowhead). HRCT: high-resolution computed tomography

He was admitted and started empirically on IV ceftriaxone. HRCT temporal bone (Figure 4) showed left foramen of Rossenmuller obliteration, no fat plane separating the left para-pharyngeal and prevertebral plane space, the clivus was eroded, and the jugular foramen widened.

His facial nerve palsy worsened, and he developed aspiration symptoms with fluids. The flexible endoscope showed sluggish movements of the left vocal cord, absent laryngeal sensations, and absent gag reflex. He deteriorated clinically with hematemesis, and his Glasgow Coma Scale (GCS) dropped. He became unconscious with asystole on the cardiac monitor. He was intubated, and cardiopulmonary resuscitation was commenced within 10 minutes. Return of spontaneous circulation was achieved.

Oesophago-gastro-duodenoscopy revealed blood clots in the fundus and erosive gastritis with gastropathy. His condition further deteriorated, and with his unstable condition, the family agreed not to proceed with active resuscitation. The patient did not survive and succumbed to death, with the cause of death being the chronic base of skull osteomyelitis with central nervous system extension.

## Discussion

We reported a typical case of skull base osteomyelitis with otogenic origin. SBO is most commonly observed in male, diabetic, and immunocompromised patients [1,4]. SBO presents initially with non-specific symptoms, for example, headaches, fever, and elevated inflammatory markers, and as a sequel of malignant otitis externa [2]. Cranial neuropathies develop on progression; therefore, its clinical picture resembles skull base malignancy. Cranial neuropathies can develop due to the sub-temporal spread of infection initially involving cranial nerve VII. Facial nerve palsy HB grade II was one of the first symptoms of SBO in our patient. The facial nerve passes through narrow bony canals and the internal acoustic meatus at the base of the skull. In osteomyelitis, the bones become infected, and foci of inflammation, erosion, and necrosis develop. In milder cases, it only causes compression of the nerve (neuropraxia) but can even progress to inflammation and erosion of the nerve (axonotmesis). In extensive skull base osteomyelitis, as was the case with our patient facial nerve involvement is usually present. 

Extension of disease to the jugular foramen causes Vernet’s syndrome, characterized by 9th, 10th, and 11th nerve palsy, as was the case with our patient (confirmed by HRCT temporal bone) [4,7,8]. Paralysis of the glossopharyngeal and vagus nerve was the cause of aspiration of liquids in our patient, as these nerves carry sensory fibers from the larynx. The absence of the gag reflex was also due to the 9th and 10th nerve palsy. Further spread to the hypoglossal foramen causes Collet Sicard’s syndrome, characterized by 9th, 10th, 11th, and 12th cranial nerve palsy [7].

In SBO, keeping a high index of clinical suspicion is crucial in making an early diagnosis. Elevated erythrocyte sedimentation rate (ESR), C-reactive protein (CRP), and leukocytosis can guide physicians toward SBO over a malignancy as the diagnosis [1,4,7]. Our patient had elevated serum inflammatory markers, which helped rule out malignancy.

Imaging plays a crucial role in the diagnosis and follow-up of SBO. Contrast-enhanced CT scan is usually the first-line imaging for any head and neck infection. It can aptly identify areas of opacification and mucosal thickening. HRCT scan and gadolinium-enhanced magnetic resonance imaging (MRI) are necessary to identify the extent of bony invasion of the infectious debris. HRCT can identify the extent of cortical involvement and highlight areas of bone demineralization. A CT angiography (CTA) or CT venography (CTV) may be considered to rule out vascular and cavernous sinus involvement [9]. MRI is the investigation of choice when evaluating the intracranial or intracerebral spread of the infection. A combination of T1, T2, diffusion-weighted (DWI) and fat-saturated images can aid clinicians in identifying the extent and possible complications of the infection [8,9]. The confirmatory test was a biopsy of the lesion. In our patient, the diagnosis of skull base osteomyelitis was confirmed on a biopsy of the left nasopharyngeal mass.

MRI is the preferred modality for follow-up of the disease. This is because the improvement in the soft tissue findings better correlates with clinical improvement as compared to the bone findings. The destructive changes in the bones may persist for months to years and may not provide an accurate prognostic report [8]. Although technetium 99 can identify infectious foci, its usage is less prevalent because of its higher cost and labor requirements when compared to alternative methods. Its capacity to identify low-grade infections is reduced. Since successfully treated infectious lesions lose their ability to concentrate the tracer, Ga-67-citrate scans typically normalize concurrently with treatment response. As a result, monitoring therapy response frequently makes use of these traces [10].

The management of SBO is guided by culture and sensitivity analysis. Long-term IV antibiotic or antifungal therapy is the mainstay of treatment [2,9,11]. In our patient, broad-spectrum IV antibiotic therapy was used and the patient had a good initial response to it. However, the prognosis was not good in our patient, presumably due to the co-morbid, i.e., uncontrolled diabetes mellitus and end-stage renal disease. Adjuvant hyperbaric oxygen therapy is an evolving treatment modality for SBO. In a retrospective cohort study, patients who were treated with hyperbaric oxygen therapy were compared with patients who were given traditional treatment. It was concluded that patients treated with hyperbaric oxygen therapy had better neurologic outcomes. Almost all patients had complete recovery of their cranial neuropathies [12].

## Conclusions

Skull base osteomyelitis is an infection in elderly, diabetic, and immunocompromised patients. Early diagnosis is challenging due to its clinical and radiological resemblance to skull base malignancies. Early diagnosis and treatment are crucial to achieve a good prognosis.
